# Positive predictive value of MRI vacuum biopsies in the diagnosis of nonmass-like lesions of the breast

**DOI:** 10.1186/bcr3252

**Published:** 2012-11-09

**Authors:** WL Teh, EK Papantoniou, F Ng

**Affiliations:** 1 Northwick Park Hospital, Harrow, UK

## Objective

To evaluate the positive predictive value of MRI scoring for malignant mass-like (ML) and nonmass-like (NML) lesions based on the BI-RADS descriptors. To identify MRI characteristics of nonmass lesions which predict malignancy for invasive and non-invasive cancers.

## Methods

Retrospective analysis of 486 MRI-guided vacuum biopsies performed at Northwick Park Hospital between April 2006 and November 2011. Each lesion was categorised according to BI-RADS lexicon (ML vs. NML lesions) and time-enhancement curves, and given an overall score of MRI 1 to 5 according to overall level of suspicion for malignant disease where MRI 4 and 5 are considered suspicious or diagnostic for malignancy. Biopsy and surgical histology results obtained.

## Results

A total of 291 ML and 152 NML lesions, of which there were 150 cancers diagnosed. Positive predictive value of MRI characteristic for malignant mass lesions is 70%. Positive predictive value of MRI characteristic for nonmass lesions is 57%. Segmental enhancement is the most common MRI morphology found in 45% DCIS. No specific features predict for invasive disease in NML lesions. Time-enhancement curves were mainly type 2 (44.6%) and type 3 (52.7%) in malignant ML lesions and unhelpful in predicting malignancy in NML lesions (72% type 2 and 59% type 3 were benign).

## Conclusion

No specific BI-RADS feature predicts for invasive disease in NML lesions. Segmental enhancement is the most common MRI appearance for DCIS. Time-enhancement curves are unhelpful in predicting malignancy in NML lesions.

